# Oral Health-Related Quality of Life and Self-Reported Oral Health Status Are Associated with Change in Self-Reported Depression Status: A Cohort Study

**DOI:** 10.3390/jcm15010376

**Published:** 2026-01-04

**Authors:** Noriko Takeuchi, Takayuki Maruyama, Naoki Toyama, Yuzuki Katsube, Takahiro Tabuchi, Daisuke Ekuni

**Affiliations:** 1 Department of Preventive Dentistry, Division of Dentistry, Medical Development Field, Okayama University, Okayama 700-8558, Japan; 2 Department of Preventive Dentistry, Faculty of Medicine, Dentistry and Pharmaceutical Sciences, Okayama University, Okayama 700-8558, Japan; t-maru@md.okayama-u.ac.jp (T.M.); pu171qxi@s.okayama-u.ac.jp (N.T.); dekuni7@md.okayama-u.ac.jp (D.E.); 3 Dental School, Okayama University, Okayama 700-8558, Japan; p37s2kfk@s.okayama-u.ac.jp; 4 Division of Epidemiology, School of Public Health, Tohoku University Graduate School of Medicine, Sendai 980-8575, Japan; tabuchitak@gmail.com

**Keywords:** oral health-related quality of life, depression status, cohort study

## Abstract

**Background/Objectives**: Oral health-related quality of life (OHRQoL) may influence mental health outcomes, yet longitudinal evidence on its association with depression remains limited. This study aimed to examine whether oral health status and OHRQoL are associated with a change in self-reported depression status among adults in Japan. **Methods**: We analyzed data from the Japan COVID-19 and Society Internet Survey (JACSIS), conducted in 2022 and 2023. A total of 15,068 participants aged ≥20 years without depression at baseline were included. Depression status was identified by self-reported measures between the two survey waves. Logistic regression models estimated odds ratios (ORs) and 95% confidence intervals (CIs) for change in self-reported depression status in relation to OHRQoL and oral health status, adjusting for sociodemographic and behavioral factors. **Results**: During follow-up, 218 participants (1.45%) reported a change in self-reported depression status. Poorer OHRQoL was significantly associated with a change in self-reported depression status (OR: 1.018; 95% CI: 1.001–1.036; *p* = 0.039). Additional risk factors included younger age (OR: 0.974; 95% CI: 0.964–0.985), participation in hobbies and cultural activities (OR: 2.224; 95% CI: 1.498–3.302), habitual use of sleeping pills or anxiolytics (current use OR: 3.512; 95% CI: 2.267–5.442), increased loneliness (OR: 1.217; 95% CI: 1.140–1.299), lower life satisfaction (OR: 0.900; 95% CI: 0.836–0.969), and poor self-rated health (OR: 2.921; 95% CI: 1.810–4.715). **Conclusions**: Impaired OHRQoL was associated with a change in self-reported depression status, potentially through psychosocial mechanisms. These findings suggest that oral health and OHRQoL may be relevant factors to consider in integrated oral and mental health approaches in clinical practice.

## 1. Introduction

Depressive disorders are recognized as a major cause of adverse health effects from early adulthood to old age [1], with over 330 million people affected worldwide [2]. In recent years, there has been increasing global concern regarding mental health issues such as depression, anxiety, and stress. According to the disability-adjusted life years metric developed by the World Health Organization, depression ranked third in 2004, is projected to rise to second by 2020, and to become the leading cause of adverse health effects by 2030 [3]. This indicates that diseases and disabilities that rank higher impose a greater socioeconomic burden on society.

The etiology of depression is complex, involving a myriad of factors ranging from biological to lifestyle-related, which poses challenges for primary prevention strategies [4]. According to de Sousa et al. [5], in their review of a longitudinal study, the course of depression varies among individuals and is influenced by factors such as age and Sex [6,7,8,9], social isolation [6,7,10], socioeconomic factors including childhood and current economic status [11], educational level [10,11,12,13,14], employment [9,13,15], chronic conditions and comorbidities [7,8,13,16,17] such as arthritis/rheumatism, asthma, stroke, coronary heart disease, mental health issues, and having more than three chronic diseases), health behaviors [18,19] such as sedentary lifestyles and smoking, self-rated health [7], and family support [20]. Additionally, factors such as participation in extracurricular activities [21], the use of sleeping medications [22], child abuse [23], spousal loss [24], social networks [21,25], event participation, decreased life satisfaction [26], and health-related quality of life (HRQOL) [27,28] have also been reported to be associated with depression.

There have also been reports of an association between depression and oral health status. Factors such as tooth loss [27,29,30,31,32], oral pain [33,34], periodontal disease [35,36,37], dental caries [38], deterioration of oral function [31], worsening oral hygiene [39], dental anxiety [40], dental visits within the past year [41], and oral health-related quality of life (OHRQOL) [42,43] have been linked to depressive symptoms. In their review, Karimi et al. [31] suggested a strong correlation between oral health habits, general health practices, and unhealthy lifestyles. These factors are considered potential causes of depression [44,45]. Therefore, they discussed the possibility not only that depression affects oral health, but also that oral health may impact depression. However, most studies have been cross-sectional, leaving many questions unanswered about how oral health impacts the development of depression.

Therefore, we hypothesized that OHRQOL and oral health status are associated with self-reported depression status over time. The aim of this cohort study was to examine the association between oral health status, OHRQOL, and change in self-reported depression status among participants without self-reported depression status at baseline.

## 2. Materials and Methods

### 2.1. Participants

This cohort study utilized data from the Japan COVID-19 and Society Internet Survey (JACSIS) a large-scale nationwide longitudinal study designed to investigate the social and health impacts of the COVID-19 pandemic in Japan. For the present analysis, we used data collected in the 2022 and 2023 survey waves, which included approximately 31,000 individuals aged 16–81 years. Details of the overall study design have been described elsewhere [46,47]. Conducted in 2022 and 2023, which surveyed 31,000 individuals aged 16–81 years. Sex was self-reported as male or female; gender identity was not assessed. Random sampling was performed based on sex, age, and prefecture, and then panel members were invited to participate in an online survey that included various questions on lifestyle, health, social interactions, and economic activities, as well as questions related to COVID-19. Participants provided web-based informed consent before answering the online survey and could withdraw consent at any time.

The survey for FY2022 started on 12 September 2022 and ended on 19 October 2022. The survey for FY2023 started on 25 September 2023 and concluded on 17 November 2023. The inclusion criteria were reporting no self-reported depression status at baseline in 2022, age 20 years or older, and providing valid responses to all question items. The exclusion criteria were having been a self-reported depression status presently or in the past, taking less than 15 min to respond, answering “yes” to all drug use items, answering “yes” to all systemic disease items, age less than 20 years, and having missing data. Sex was included as a covariate in all statistical analyses. The study protocol was approved by the Osaka International Cancer Institute Research Ethics Committee (approved on 19 June 2020; approval No. 20084) as well as the Ethics Review Board of Okayama University (approval Nos.: 2403-044, 2408-009).

### 2.2. Measures

#### 2.2.1. Depression Status

We asked the participants, “Do you currently have depression?”, to which they were instructed to respond with one of the following options:

(1) “Never had it”;
(2) “Not currently depressed but have had it in the past”;
(3) “Currently have depression (under treatment with medication)”;
(4) “Currently have depression (under treatment without medication)”;
(5) “Currently have depression (not under treatment)”.

Responses indicating (3), (4), or (5) were classified as “self-reported depression status present”. As this measure does not capture the clinical onset or progression of depression, we refer to the outcome as a change in self-reported depression status between baseline and follow-up.

#### 2.2.2. Oral Health-Related Quality of Life (OHRQOL)

Many studies have shown that OHRQOL is influenced by factors such as the number of teeth, the presence of dentures, the need for dental treatment, and the awareness of oral dryness [48,49,50,51]. Among the evaluation indicators for OHRQOL, the Oral Health Impact Profile (OHIP) and the General Oral Health Assessment Index (GOHAI) have been translated into Japanese and are frequently used. However, while the GOHAI is affected by functional disabilities and pain, the OHIP is influenced by psychosocial factors [48,50]. Therefore, in this study, we used the Japanese version of the OHIP-14, a short version of the OHIP (see Table 1 for the English version), as a measure of OHRQOL.

The OHIP-14 consists of 14 questions, each with five response options regarding experiences within the past month. The respondents were asked to select the option that best applied to them, with the scoring as follows: “Always” = 4, “Often” = 3, “Sometimes” = 2, “Rarely” = 1, and “Never” = 0. The OHIP-14 total score is calculated by summing the scores from each question, with a maximum possible score of 56. A higher score indicates poorer OHRQOL.

#### 2.2.3. Oral Health Status

Oral health status was assessed for tooth loss, periodontal disease and oral pain.

##### Tooth Loss

We asked the participants, “Have you ever had permanent teeth extracted because of cavities or periodontal disease? If so, please specify the number of teeth extracted (excluding teeth extracted because of an external cause such as injury or orthodontic treatment)”. They were instructed to respond with one of the following options: “0 teeth, 1 tooth, 2 teeth, 3 teeth, 4 teeth, 5 teeth, 6–9 teeth, 10–19 teeth, 20–27 teeth, or 28 teeth or more (edentulous)”. Responses were categorized into three groups: 0–5 teeth, 6–9 teeth, and 10 or more teeth.

##### Periodontal Disease Screening

For the assessment of periodontal status, we used the periodontal screening index developed by Yamamoto et al. [52]. We asked the participants the following questions: “Do you experience bleeding from your gums (such as when brushing your teeth)?”, “Do you feel that your gums have receded compared with before, making your teeth appear longer?”, “Have you ever been told by a dental clinic that you need treatment for periodontal disease or gum issues?”, and “Are you currently a smoker or have you smoked in the past?” Participants who answered “Yes” to three or more of these questions were classified as “having periodontal disease”.

##### Oral Pain

We asked the participants, “Have you experienced tooth pain because of cavities in the past 2 months?” and “Have you experienced gum pain because of periodontal disease in the past 2 months?” They were instructed to answer “Yes” or “No”. Participants who answered “Yes” to one or more of these questions were classified as having “oral pain”.

##### Oral Health Behavior

We also asked the participants, “Have you visited a dentist in the past year?” They were instructed to respond with either “Yes” or “No”.

#### 2.2.4. Covariates

##### Developmental Factors

As developmental factors, we evaluated childhood socioeconomic status and experiences of abuse during childhood.

Regarding childhood socioeconomic status, we asked the participants, “Did you experience financial hardship before turning 18?” [53]. They were instructed to respond with “Yes” or “No”.

Regarding childhood abuse, we asked the following questions [53]: “Have you ever been injured by being severely beaten by a parent before turning age 18?”, “Have you ever lacked necessary care, such as meals or clothing, before turning age 18?”, “Has a parent ever said hurtful or insulting things to you before turning age 18?”, and “Did you feel suffocated because your opinions were never respected by your parents before turning age 18?”. The participants were instructed to answer “Yes” or “No” to each question. Those who answered “Yes” to one or more questions were classified as having experienced abuse. These variables were included as background factors because early-life adversity may influence stress vulnerability [54], health behaviors [55], and oral health [56,57], which are relevant to depression risk even in adulthood.

##### Sociodemographic and Relationship Characteristics

We also evaluated age, sex, years of education, and annual income. We classified annual income into the following categories: “Less than 3 million yen”, “3 million yen to less than 6 million yen”, “6 million yen to less than 9 million yen”, and “9 million yen or more” [58].

We inquired about educational background (junior high school, high school, vocational school, junior college, university, and graduate school) and assigned the following years of education: “Junior high school” = 9 years, “High school” = 12 years, “Vocational school” = 13 years, “Junior college” = 14 years, “University” = 16 years, and “Graduate school” = 18 years [59]. Responses that fell outside these categories or were marked as “unknown” were excluded from the analysis.

##### Lifestyle Factors

Regarding lifestyle factors, we evaluated participation in extracurricular activities, physical activity, sitting for more than 240 min per day, smoking history, and excessive alcohol intake.

We asked the participants about extracurricular activities and light exercise with the following questions: “Do you participate in hobby, learning, or educational groups or clubs?” and “Do you engage in walking or equivalent physical activity for more than 1 h per day in your daily life?”. The participants were instructed to respond with “Yes” or “No”.

Regarding sitting for more than 240 min per day, we asked the participants, “What was your average sitting time per day in the past month?”. Those who reported sitting for more than 240 min were classified as sitting for over 240 min per day. The cutoff of 240 min was based on previous evidence indicating that prolonged sedentary behavior is associated with adverse health outcomes, including increased risk of type 2 diabetes and early mortality [60], and depressive symptoms in older adults [22].

Regarding smoking history, we asked the participants to respond with one of the following options: “Currently smoking”, “I used to smoke but do not smoke now”, or “I have never smoked”.

Regarding excessive drinking, daily alcohol consumption was converted into sake, with the threshold set at more than 3 gou (a traditional Japanese measurement for sake) per day for men and more than 2 gou per day for women [61].

##### Physical Health Status

We evaluated physical health status by assessing the number of diseases and the habitual use of sleeping pills and antianxiety medications.

We assessed the number of diseases by asking about the current prevalence of the following conditions: hypertension, diabetes, hyperlipidemia, pneumonia/bronchitis, asthma, heart disease, cerebrovascular disease, chronic obstructive pulmonary disease, kidney disease, liver disease, immune disorders, and cancer/malignant tumors. The total number of diseases for which the participants answered “currently present” was classified as the number of diseases.

With regard to the habitual use of sleeping pills, the participants were asked whether they “currently use sleeping pills”, “never used them”, “used them at least once but not habitually”, “used them habitually but not anymore”, “used them occasionally some days”, or “used them almost every day”. Respondents who answered “never used them” or “used them at least once but not habitually” were defined as “never used them habitually”, and those who answered “used them occasionally on some days” or “used them almost every day” were defined as “used them habitually”.

##### Psychosocial Factors

Regarding psychosocial factors, we assessed the following: loss of a spouse, family relationships, neighborhood, conversation with nonliving family members, frequency of eating alone (less than once a week, 1–5 times a week, 6–7 times a week), loneliness, social networks, risk of isolation, HRQOL, and life satisfaction.

We used social capital in terms of the family to describe familial relationships [62]. We asked the participants to respond to the following items: “I like my family”, “I enjoy spending time with my family”, “I go to someone in my family when I have a problem”, “I trust my family”, “My family goes on summer holidays and celebrates birthdays together”, “My family follows family rules”, and “We all help each other when someone in my family has a problem”. The participants were asked to rate each of these items as follows: “Strongly agree” (1 point), “Somewhat agree” (2 points), “Neither agree nor disagree” (3 points), “Disagree” (4 points) and “Strongly disagree” (5 points). The total score was used as the family social capital score.

The feeling of loneliness was assessed using the Japanese version of the UCLA Loneliness Scale Version 3, 3-item Short Form (UCLA-LS3-SF-3) [63,64]. The question items on the UCLA-LS3-SF-3 are each rated on a 4-point Likert-type scale, ranging from “Always” to “Never”, with scores assigned from 1 to 4. The total score is then calculated (range: 3–12 points), with higher scores indicating a greater sense of loneliness.

To evaluate social networks, we utilized the Japanese version of the Lubben Social Network Scale (LSNS-6) [65,66], a globally recognized screening tool for social isolation among older adults. The LSNS-6 measures the size of a participant’s active and intimate network with family and friends, assessing their ability to discuss issues with or seek help from others. Each item on the LSNS-6 is scored from 0 to 5 points. The total score is the evenly weighted sum of these six questions, with a range from 0 to 30 points. Higher scores indicate a larger social network. A score below 12 points is considered to indicate a greater risk of social isolation [67]. Therefore, a score below 12 points was categorized as an increased risk for social isolation.

The assessment of HRQOL utilized the HRQOL-4 developed by the Centers for Disease Control and Prevention (CDC; hereafter referred to as CDC HRQOL-4) [68,69]. The four items on the CDC HRQOL-4 assess the following: self-rated health; number of unhealthy days due to physical illness or an injury during the past 30 days; number of unhealthy days due to stress; depression, and problems with emotions during the past 30 days; and number of days with limitations in self-care, work, or recreation due to poor physical or mental health during the past 30 days. We dichotomized each CDC HRQOL-4 component variable as good vs. poor: general health into good (good or very good) vs. poor (poor or fair) [70], physical and mental health into good (<14 days/month) vs. poor (≥14 days/month), and activity limitations into good (<14 days/month) vs. poor (≥14 days/month) [70]. The number of “poor” values from four CDC HRQOL-4 components was summed, and then the overall HRQOL variable was dichotomized into good (none) vs. poor (one or more). The four dichotomized variables were then summed (range 0–4) and further dichotomized as “good health” if scored from 0 to 2 and “poor health” if scored 3–4 [71].

Regarding life satisfaction, the participants were asked, “How satisfied are you with your overall life recently?”. Responses were given on a Likert scale ranging from 0 (not applicable at all) to 10 (completely applicable).

### 2.3. Statistical Analysis

Among individuals who reported no self-reported depression status in the 2022 survey, those who reported a self-reported depression status in the 2023 survey were classified as the “change in self-reported depression status” group, whereas those who continued to report no self-reported depression status were classified as the “no change in self-reported depression status” group. Associations between outcome status and covariates were analyzed using the chi-square test and Student’s *t*-test. Variable selection was based on the biopsychosocial model of depression and supported by previous studies [53,54,55,56,57,58,59,60,61,62,63,64,65,66,67,68,69,70,71]. Items with a *p*-value < 0.1 were included as independent variables, and change in self-reported depression status was used as a dependent variable in a multivariate binary logistic regression analysis. Sex (male/female) was included as a covariate in all models. Model fit was evaluated using the Hosmer–Lemeshow goodness-of-fit test, ROC curve analysis (AUC), and pseudo R^2^ (Cox and Snell and Nagelkerke). Multicollinearity was assessed by calculating variance inflation factors (VIF) from an auxiliary linear regression model using the same predictors, as VIF is not directly available for logistic regression. A VIF < 10 was considered acceptable. In addition to logistic regression models, we conducted mediation analyses using structural equation modeling (SEM) to examine whether baseline psychosocial factors mediated the association between baseline OHIP-14 scores and change in self-reported depression status. We specified structural pathways from the OHIP-14 to psychosocial factors and to change in self-reported depression status, allowing for partial mediation. Indirect effects, direct effects, and total effects were estimated as nonlinear combinations of model parameters using the delta method, with 95% confidence intervals reported. SEM was estimated using maximum likelihood (ML) estimation. Model fit was assessed using RMSEA, Tucker–Lewis Index (TLI), and Comparative Fit Index (CFI), with conventional thresholds (RMSEA < 0.05, TLI and CFI ≥ 0.90) indicating acceptable fit. IBM SPSS Statistics, version 26.0, IBM Japan, Tokyo, Japan) and STATA/SE version 19.0 (StataCorp LLC, College Station, TX, USA) was used for all statistical analyses, with the level of significance set at 5%.

### 2.4. AI Software Disclosure

ChatGPT (GPT-3.5, OpenAI, San Francisco, CA, USA) was used as an ancillary tool to verify the coherence and logical consistency of the manuscript. The tool was not employed for generating scientific content, data analysis, or interpretation. All suggestions provided by the AI were critically reviewed and accepted or rejected at the authors’ discretion.

## 3. Results

**Figure 1:**
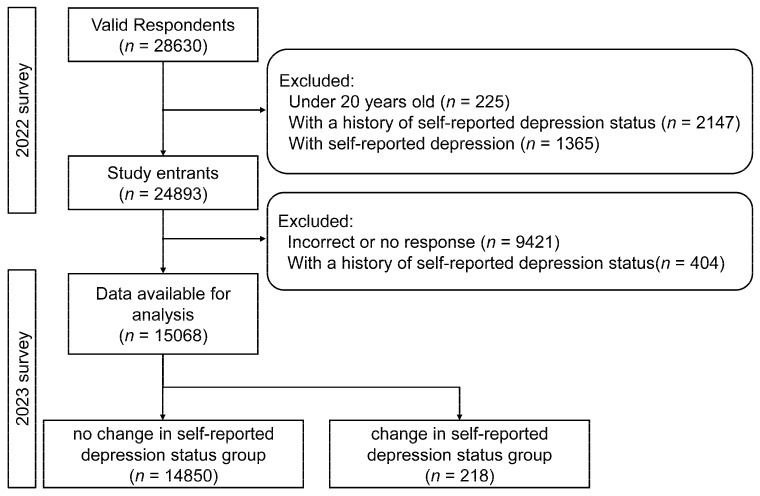
Flowchart of the study. Flowchart illustrating participant selection for the 2022 baseline survey and 2023 follow-up, including applied inclusion and exclusion criteria and the final sample of 15,068 participants for analysis.

Figure 1 shows the flowchart of the study. Flowchart illustrating the participant selection process for the study conducted in 2022 and 2023. The diagram shows inclusion and exclusion criteria applied to respondents from the 2022 survey (*n* = 28,630) and the subsequent 2023 follow-up. Participants under 20 years old, those with a history of depression, or those with depression at baseline were excluded, resulting in 24,894 entrants. Further exclusions for incorrect or missing responses and history of depression during follow-up yielded 15,068 participants for analysis. These were categorized into two groups: without change in self-reported depression status (*n* = 14,850) and with change in self-reported depression status (*n* = 218). Abbreviations: *n* = number of participants.

Table 2 shows the characteristics of the respondents, including sex distribution (male/female). The change in the self-reported depression status group consisted of 218 individuals (1.5%). The results of the comparison between participants with and without a change in self-reported depression status are shown in Table 3. The items that differed between the two groups at a screening level (*p* < 0.1) included dental visits within the past year, OHIP-14 score, age, sex, income, smoking history, participation in hobbies, learning, and cultural activities, walking or equivalent physical activity for more than one hour per day, chatting with non-cohabiting family members, economic situation until age 18, abuse until age 18, frequency of eating alone, habitual use of sleeping pills or anxiolytic medications, family social capital, UCLA-LS3-SF-3 score, risk of social isolation, life satisfaction, and CDC HRQOL-4 score.

Table 4 shows the results of the multivariate binomial logistic regression analysis, with these items included as independent variables and change in self-reported depression status as the dependent variable. After adjusting for other covariates, the items significantly associated with change in self-reported depression status were: “OHIP-14: (odds ratio [OR]: 1.02, 95% confidence interval [CI]: 1.00–1.04, *p* = 0.039)”, “Age (OR: 0.97, 95% CI: 0.96–0.99, *p* < 0.001)”, “Participation in hobbies, learning, and cultural activities: Yes (OR: 2.22, 95% CI: 1.50–3.30, *p* < 0.001)”, “Habitual use of sleep or antianxiety medications: Ever (OR: 1.97, 95% CI: 1.06–3.68, *p* = 0.033) and Currently (OR 3.51, 95% CI: 2.27–5.44, *p* < 0.001)”, “UCLA-LS3-SF-3 (OR: 1.22, 95%CI: 1.14–1.30, *p* < 0.001)”, “Life satisfaction (OR: 0.90, 95% CI: 0.84–0.97, *p* = 0.005)”, and “CDC HRQOL-4: Poor (OR: 2.92: 95% CI: 1.81–4.72, *p* < 0.001)”. The model demonstrated good fit (Hosmer–Lemeshow χ^2^ = 9.445, *p* = 0.306), moderate explanatory power (Nagelkerke R^2^ = 0.153), and good discrimination (AUC = 0.807, 95% CI: 0.774–0.840).

**Figure 2:**
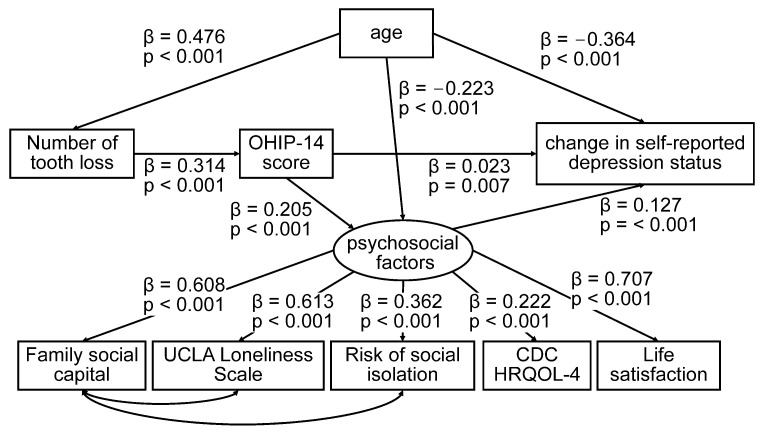
Structural equation model illustrating hypothesized mediation pathways among oral health-related quality of life (OHIP-14), psychosocial factors, and change in self-reported depression status. This model includes direct and indirect pathways, suggesting that psychosocial factors may mediate the association between OHIP-14 and change in self-reported depression status. Standardized path coefficients (β) and *p*-values are shown for each relationship. The model demonstrated good fit (RMSEA = 0.039, CFI = 0.969, TLI = 0.949). Abbreviations: OHIP-14, Oral Health Impact Profile-14; UCLA, University of California, Los Angeles; CDC HRQOL-4, Centers for Disease Control and Prevention Health-Related Quality of Life-4.

The SEM results are shown in Figure 2. The model demonstrated a good fit to the data (RMSEA = 0.039, CFI = 0.969, TLI = 0.949). Baseline OHIP-14 scores were associated with subsequent change in self-reported depression status, both directly and indirectly through psychosocial factors, including family social capital, UCLA Loneliness Scale, risk of social isolation, CDC HRQOL-4, and life satisfaction, which were modeled as latent variables. The direct effect of OHIP-14 on change in self-reported depression status was β = 0.00039, z = 2.69, *p* = 0.007; 95% CI: 0.00011–0.00068. The indirect effect of OHIP-14 on change in self-reported depression status via psychosocial latent factors was β = 0.00045 (z = 10.89, *p* < 0.001; 95% CI: 0.00037–0.00053). The total effect was β = 0.00084 (z = 5.95, *p* < 0.001; 95% confidence interval: 0.00057–0.00112).

## 4. Discussion

In this study, we examined change in self-reported depression status and its associated factors among adults aged 20 years and older. The results showed that younger age, participation in hobbies, learning, and cultural activities, habitual use of sleeping pills and anxiolytics, lower scores on the UCLA-LS3-SF-3 (indicating feelings of loneliness), higher scores on the CDC HRQOL-4 (indicating poor self-rated health), lower life satisfaction scores (indicating low satisfaction), and higher scores on the OHIP-14 (indicating poor OHRQOL) were significantly associated with change in self-reported depression status. These results suggest that maintaining good OHRQOL is associated with self-reported depression status.

In this study, a significant association was observed between OHRQOL and change in self-reported depression status. Possible mechanism linking poorer OHRQOL with change in self-reported depressive status may involve reduced social activities and psychosocial difficulties. Ohi et al. [27] examined whether OHRQOL impairment at baseline was associated with the development of depressive symptoms 4 years later among participants without depressive symptoms by conducting a questionnaire survey on adults aged 55 years and older. They reported that baseline OHRQOL impairment was significantly associated with an increased risk of depressive symptoms, independent of potential confounding factors such as dental status and dental visits. Those findings suggest that a deterioration of social activities and psychosocial issues may be involved in the pathway from OHRQOL impairment to the development of depressive symptoms. Similarly, Zhang et al. [72] reported in a cross-sectional study that college students with inadequate OHRQOL are more likely to exhibit depressive symptoms. Their findings suggest that OHRQOL may influence the prevalence of depressive symptoms through a decrease in self-esteem and life satisfaction caused by barriers to interpersonal communication. These previous reports investigated the relationship between self-assessment depression scales and OHRQOL. By contrast, the present study examined the association between change in self-reported depression status and OHRQOL. In the present study, OHRQOL was associated with change in self-reported depression status in conjunction with psychosocial factors such as social activities, psychosocial problems, impaired interpersonal communication, and decreased self-esteem and life satisfaction.

In this study’s mediation analysis, psychosocial factors were found to partially mediate the association between reduced oral health-related quality of life and change in self-reported depression status, although the direct effect remained significant. This suggests that oral health may be associated with mental health both directly and indirectly through psychosocial pathways such as loneliness and reduced social capital. Sung et al. [73] reported that social participation mediated the pathway from oral-related quality of life (General Oral Health Assessment Index) to depressive symptoms (The Center for Epidemiologic Studies Depression Scale) in older adults aged 65 to 95 years using a cross-lagged panel model. Although the subjects and assessment methods differ, this finding is considered to support the present study. However, the relationship between OHRQOL and depression may be bidirectional. Depression can cause behavioral changes such as neglecting oral hygiene or avoiding dental treatment [74], which may worsen oral health over time and potentially lead to a decline in OHRQOL. Therefore, while a decline in OHRQOL may be a contributing factor to depression status, depression itself may also be associated with a decline in OHRQOL. In this study, since oral health-related QOL was not assessed at re-evaluation, it remains unclear whether self-reported depression status is associated with oral health-related QOL. To clarify the causal relationship in this association, longitudinal studies that repeatedly measure both OHRQOL and depression are necessary.

No significant association was found between missing teeth and change in self-reported depression status. Kusama et al. [75] conducted a 3-year longitudinal study involving participants with an average age of 72.7 years to investigate the relationship between tooth loss and depressive symptoms. They found that having 19 or fewer teeth significantly increased the risk of depressive symptoms by 1.3 times. This relationship was notably mediated by difficulties in speaking, smiling, and chewing. Ohi et al. [27] examined the association between depressive symptoms and the number of teeth among Japanese older individuals aged 55 years and older. While a cross-sectional study revealed a correlation, a 4-year longitudinal study did not. Chu et al. [29] reported a connection between the number of teeth and the onset of depression in a 20-year cohort study with an average participant age of 58.8 years. They considered the potential involvement of nutritional status and social participation in this relationship. This may be because the study period was short and the study population did not comprise only older adults.

In the present study, no significant association was found between periodontal disease screening and change in self-reported depression status. In an 11-year cohort study, Hsu et al. [76] found that periodontitis (International Classification of Diseases, Ninth Revision, Clinical Modification [ICD-9-CM] 523.4x and 523.5x) was associated with the onset of depression (ICD-9-CM 296.2x, 296.3x, 300.4x, and 311.xx). They considered that periodontitis leads to the release of proinflammatory cytokines such as interleukin (IL)-1β, IL-6, and tumor necrosis factor into systemic circulation, and that psychological stress in patients with periodontitis promotes disturbances in the hypothalamic–pituitary–adrenal (HPA) axis and related hypercortisolism. This, in turn, affects immune dysfunction and neuroinflammation, potentially leading to the development of depression. In the present study, the lack of an association may be due to the use of questionnaire-based screening indicators rather than oral examinations for periodontal disease. Additionally, the short follow-up period of 1 year may have contributed to the absence of observed associations.

In a systematic review, Anita et al. [34] suggested a correlation between oral-facial pain (e.g., temporomandibular joint pain, stomatitis) and depression. It is noted that oral-facial pain activates the HPA axis, which is involved in the release of cortisol hormones in patients with depressive symptoms. In this study, only the presence or absence of pain related to caries and periodontal disease was investigated, and temporomandibular joint pain was not included, which may explain the lack of observed associations.

In this study, no significant association was observed between regular dental visits and change in self-reported depression status. In a cross-sectional survey, Peltzer et al. [41] reported a correlation between depression and regular dental visits. Although they did not discuss the mechanisms, it is generally considered that lower socioeconomic status can lead to poorer oral health behaviors. It has also been suggested that university students with lower socioeconomic status may be less likely to attend regular dental check-ups because of their inability to afford dental treatment [77]. In the present study, a significant association was found between income and the presence of regular dental visits, but neither income nor the presence of regular dental visits was associated with change in self-reported depression status.

This study was based on a large web-based survey of residents in 47 prefectures throughout Japan, which is a strength in ensuring high generalizability. Furthermore, the large sample size from the JACSIS is a strength of this study.

The change in self-reported depression status in this study was 1.45%. Büchtemann et al. [78] reported in a systematic review of elderly individuals aged 70 and older that the incidence of major depression ranged from 1.7% to 7.6%. Although the definitions of depression and the age groups of the study populations differ, it cannot be conclusively stated that the incidence of depression status in this population is low.

Adverse childhood experiences are typically examined within the frameworks of life course theory and cumulative risk theory. We included these factors as background variables because adverse childhood experiences influence stress vulnerability [54], health behaviors [55], and oral hygiene [56,57], all of which are associated with depression risk in adulthood. However, their impact on short-term development should be interpreted with caution. As a sensitivity analysis, we conducted an additional binomial logistic regression including participants with a history of depression. The association between OHIP-14 scores and changes in self-reported depression status remained statistically significant, indicating that the main findings were robust.

### Limitations

This study has several limitations. First, it relied entirely on self-reporting and was not an objective survey. As a result, the actual outcomes may have been either underestimated or overestimated. Second, there are confounding factors that could not be surveyed. Genetic factors and stress resilience have not been investigated. Third, due to limitations regarding the statistical analysis, binary variables were used for exposure, mediators, and outcomes, which may have led to an overestimation or underestimation of the results. Fourth, because this study depended on an online survey, there is a possibility that the sample was overrepresented by individuals with high Internet literacy [79]. This may have led to selection bias; however, it has been confirmed that this population is not disproportionate compared with census data. In future research, objective measurements such as oral examinations and the use of medical records should be used in addition to self-reported evaluations. Fifth, this study uses a cohort study design with a one-year follow-up period, but due to the short follow-up period, there are limitations to inferring causality. Sixth, self-reported classifications were used to assess depression rather than validated symptom-based scales. While this classification considered current status and treatment, it may not have adequately reflected the episodic and recurrent nature of depression. Furthermore, although treatment information was collected, it was not included in the analysis. Seventh, OHIP-14 and depression status share psychosocial dimensions, which may lead to conceptual overlap. Therefore, the observed association should be interpreted with caution. Eighth, although the cohort study design established a temporal sequence by excluding participants with baseline depression status, caution is warranted in interpreting the association between OHIP-14 and self-reported depression status. This exclusion prevented us from examining life-course pathways through which early-life adversities or psychosocial vulnerability may influence later depression. Furthermore, several covariates included in the fully adjusted models (e.g., use of sleep medication, loneliness, life satisfaction) conceptually overlap with depressive affect, which may limit the interpretability of the adjusted associations. Since both measures capture subjective aspects of overall health status, their correlation may reflect conceptual overlap rather than a causal pathway. Furthermore, depression at follow-up was measured using a self-report item, which does not allow differentiation clinically diagnosed depression from mild symptoms or transient mood fluctuations.

## 5. Conclusions

In a cohort study of participants who self-reported no depression status at baseline, we examined whether OHRQOL and oral health status were associated with change in self-reported depression status. The results, based on self-reported data, indicate that poorer OHRQOL is associated with change in self-reported depression status, even after adjusting for other related factors. Additionally, mediation analysis using SEM suggested that psychosocial factors partially mediated this association.

## Figures and Tables

**Table 1:** English version of the Oral Health Impact Profile-14 (OHIP-14) questionnaire. OHIP-14 assesses oral health-related quality of life across 14 items. Participants responded based on experiences during the past 7 days. Response options: 0 = Never, 1 = Hardly ever, 2 = Occasionally, 3 = Fairly often, 4 = Very often.

Questionnaire Items
For the past 7 days, have you…
…had trouble pronouncing any words because of problems with your teeth or mouth? …felt that your sense of taste has worsened because of problems with your teeth or mouth? …had painful aching in your mouth? …found it uncomfortable to eat any foods because of problems with your teeth or mouth? …been self-conscious because of your teeth or mouth? …felt tense because of problems with your teeth or mouth? …had to interrupt meals because of problems with your teeth or mouth? …found it difficult to relax because of problems with your teeth or mouth? …been a bit embarrassed because of problems with your teeth or mouth? …been a bit irritable with other people because of problems with your teeth or mouth? …had difficulty doing your usual jobs because of problems with your teeth or mouth? …felt that life in general was less satisfying because of problems with your teeth or mouth …been totally unable to function because of problems with your teeth or mouth? Has been your diet been unsatisfactory because of problems with your teeth of mouth?

**Table 2:** Characteristics of participants at baseline (*n* = 15,068). Baseline characteristics of study participants, including oral health-related variables (OHIP-14 score, oral health status), sociodemographic factors, and behavioral covariates. Continuous variables are presented as mean ± standard deviation (SD), and categorical variables as *n* (%). OHIP: Oral Health Impact Profile; UCLA: University of California, Los Angeles; CDC HRQOL-4: Centers for Disease Control and Prevention Health-Related Quality of Life.

Variables	Categories	*n* (%)/Average ± SD
OHIP-14 score *		3.8 ± 6.9
Oral health status		
Number of tooth loss ^†^	None	7553 (50.1)
	1~5	6903 (45.8)
	10 or more	612 (4.1)
Periodontal disease	None	13,346 (88.6)
	Yes	1722 (11.4)
Oral pain	None	13,379 (88.8)
	Yes	1689 (11.2)
Dental visits within 1 year	Yes	9010 (59.8)
	None	6058 (40.2)
Covariates		
Age (years)		51.9 ± 16.8
Sex	Male	7715 (51.2)
	Female	7353 (48.8)
Years of Education (years)		14.4 ± 2.0
Annual income	<3 million yen	2560 (21.0)
	3–6 million yen	4819 (39.6)
	6–9 million yen	2631 (21.6)
	>9 million yen	2155 (17.7)
Smoking history	Never	9439 (62.6)
	Past	3058 (20.3)
	Currently	2571 (17.1)
Excessive alcohol intake	None	13,795 (91.6)
	Yes	1273 (8.4)
Number of diseases		0.6 ± 1.0
Participation in hobbies, study, and culture-related activities	Nonparticipation	13,112 (87.0)
	Participation	1956 (13.0)
Walking ≥ 1 h/day or equivalent physical activity	Yes	6096 (40.5)
	No	8972 (59.5)
Relationship with Neighbors	None	8338 (55.3)
	Yes	6730 (44.7)
Chatting/mingling with non-family members	None	2962 (19.7)
	Yes	12,106 (80.3)
Sitting ≥ 240 min/day	Yes	5639 (38.7)
	None	8924 (61.3)
Loss of spouse	None	13,627 (90.4)
	Yes	1441 (9.6)
Financial situation up to age 18	Not poor	11,852 (78.7)
	Poor	3216 (21.3)
Abuse experience up to age 18	None	12,606 (83.7)
	Yes	2462 (16.3)
Frequency of eating alone	<1 time/week	6005 (39.9)
	1–5 times/week	4768 (31.6)
	6–7 times/week	4295 (28.5)
Habitual use of sleeping pills/anxiolytics	No	13,911 (92.3)
	Past use	354 (2.3)
	Current use	803 (5.3)
Family social capital		14.6 ± 6.1
UCLA Loneliness Scale (3-item)		5.4 ± 2.6
Risk of social isolation	None	8686 (57.6)
	Yes	6382 (42.4)
CDC HRQOL-4	Good	14,650 (97.2)
	Poor	418 (2.8)
Life satisfaction		6.4 ± 2.3

*: average ± SD, ^†^: *n* (%).

**Table 3:** Baseline characteristics and oral health-related variables by change in self-reported depression status. Baseline characteristics and oral health-related variables are compared between participants with and without a change in self-reported depression status during follow-up. Continuous variables are presented as mean ± standard deviation (SD), and categorical variables as *n* (%). Statistical tests: Student’s *t*-test for continuous variables (‡) and Chi-square test for categorical variables (§). OHIP: Oral Health Impact Profile; UCLA: University of California, Los Angeles; CDC HRQOL-4: Centers for Disease Control and Prevention Health-Related Quality of Life.

		No Change in Self-Reported Depression Status Group (*n* = 14,850)	Change in Self-Reported Depression Status Group (*n* = 218)	*p* Value
OHIP-14 score *		3.7 ± 6.9	6.0 ± 9.0	<0.001 ^‡^
Oral health status				
Number of tooth loss ^†^	None	7437 (50.1)	116 (53.2)	0.471 ^§^
	1~5	6807 (45.8)	96 (44.0)	
	10 or more	606 (4.1)	6 (2.8)	
Periodontal disease	None	13,157 (88.6)	189 (86.7)	0.393 ^§^
	Yes	1693 (11.4)	29 (13.3)	
Oral pain	None	13,193 (88.8)	186 (85.3)	0.101 ^§^
	Yes	1657 (11.2)	32 (14.7)	
Dental visits within 1 year	Yes	8894 (59.9)	116 (53.2)	0.051 ^§^
	None	5956 (40.1)	102 (46.8)	
Covariates				
Age (years)		52.0 ± 16.8	43.9 ± 15.3	<0.001 ^‡^
Sex	Male	7590 (51.1)	125 (57.3)	0.076 ^§^
	Female	7260 (48.9)	93 (42.7)	
Years of Education (years)		14.4 ± 2.0	14.2 ± 2.1	0.262 ^‡^
Annual income	<3 million yen	2513 (21.0)	47 (26.6)	0.082 ^§^
	3–6 million yen	4746 (39.6)	73 (41.2)	
	6–9 million yen	2605 (21.7)	26 (14.7)	
	>9 million yen	2124 (17.7)	31 (17.5)	
Smoking history	Never	9317 (62.7)	122 (56.0)	0.009 ^§^
	Past	3016 (20.3)	42 (19.3)	
	Currently	2517 (16.9)	54 (24.8)	
Excessive alcohol intake	None	13,597 (91.6)	198 (90.8)	0.724 ^§^
	Yes	1253 (8.4)	20 (9.2)	
Number of diseases		0.6 ± 1.0	0.8 ± 1.6	0.960 ^‡^
Participation in hobbies, study, and culture-related activities	Nonparticipation	12,943 (87.2)	169 (77.5)	<0.001 ^§^
	Participation	1907 (12.8)	49 (22.5)	
Walking ≥ 1 h/day or equivalent physical activity	Yes	6025 (40.6)	71 (32.6)	0.019 ^§^
	No	8825 (59.4)	147 (67.4)	
Relationship with Neighbors	None	8205 (55.3)	133 (61.0)	0.100 ^§^
	Yes	6645 (44.7)	85 (39.0)	
Chatting/mingling with non-family members	None	2902 (19.5)	60 (27.5)	0.004 ^§^
	Yes	11,948 (80.5)	158 (72.5)	
Sitting ≥ 240 min/day	Yes	5549 (38.7)	90 (43.3)	0.174 ^§^
	None	8806 (61.3)	118 (56.7)	
Loss of spouse	None	13,428 (90.4)	199 (91.3)	0.805 ^§^
	Yes	1422 (9.6)	19 (8.7)	
Financial situation up to age 18	Not poor	11,695 (78.8)	157 (72.0)	0.018 ^§^
	Poor	3155 (21.2)	61 (28.0)	
Abuse experience up to age 18	None	12,456 (83.9)	150 (68.6)	<0.001 ^§^
	Yes	2394 (16.1)	68 (31.2)	
frequency of eating alone	<1 time/week	5949 (40.1)	56 (25.7)	<0.001 ^§^
	1–5 times/week	4683 (31.5)	85 (39.0)	
	6–7 times/week	4218 (28.4)	77 (35.3)	
Habitual use of sleeping pills/anxiolytics	No	13,749 (92.6)	162 (74.3)	<0.001 ^§^
	Past use	340 (2.3)	14 (6.4)	
	Current use	761 (5.1)	42 (19.3)	
Family social capital		14.6 ± 6.1	17.3 ± 6.9	<0.001 ^‡^
UCLA Loneliness Scale (3-item)		5.4 ± 2.6	7.7 ± 3.0	<0.001 ^‡^
Risk of social isolation	None	6322 (42.6)	60 (27.5)	<0.001 ^§^
	Yes	8528 (57.4)	158 (72.5)	
CDC HRQOL-4	Good	14,468 (97.4)	162 (83.5)	<0.001 ^§^
	Poor	382 (2.6)	36 (16.5)	
Life satisfaction		6.4 ± 2.3	4.6 ± 2.8	<0.001 ^‡^

*: Mean ± SD; ^†^: *n* (%); ^‡^: Student’s *t*-test; ^§^: Chi-square test.

**Table 4:** Binomial logistic regression analysis with change in self-reported depression status as dependent variable. Odds ratios (ORs) and 95% confidence intervals (CIs) for factors associated with self-reported depression status, adjusted for sociodemographic and behavioral covariates. OHIP = Oral Health Impact Profile; UCLA = University of California, Los Angeles; CDC HRQOL-4 = Centers for Disease Control and Prevention Health-Related Quality of Life; Ref = Reference category. Model fit: Hosmer–Lemeshow χ^2^ = 9.445 (*p* = 0.306); Nagelkerke R^2^ = 0.153; AUC = 0.807 (95% CI: 0.774–0.840). VIF = Variance Inflation Factor; values < 10 indicate acceptable multicollinearity.

Independent Variable	Category	Odds Ratio	95% CI	*p*-Value	VIF
OHIP-14 score	—	1.02	1.00–1.04	0.039	1.087
Dental visits within 1 year	Yes (Ref)	—			1.049
	None	1.17	0.85–1.60	0.329	
Age (years)	—	0.97	0.96–0.99	<0.001	1.246
Sex	Male (Ref)	—			1.218
	Female	1.08	0.78–1.52	0.637	
Annual income	>9 million yen (Ref)	—			1.139
	6–9 million yen	0.64	0.38–1.10	0.106	
	3–6 million yen	0.97	0.62–1.51	0.883	
	<3 million yen	1.07	0.65–1.77	0.787	
Smoking history	Never (Ref)	—			1.177
	Past	1.28	0.83–1.96	0.268	
	Current	1.28	0.86–1.92	0.221	
Participation in hobbies, study, and culture-related activities	Nonparticipation (Ref)	—			1.045
	Participation	2.22	1.50–3.30	<0.001	
Walking ≥ 1 h/day or equivalent physical activity	Yes (Ref)	—			1.073
	No	0.96	0.69–1.32	0.783	
Chatting/mingling with non-family members	None (Ref)	—			1.169
	Yes	1.05	0.72–1.55	0.788	
Financial situation up to age 18	Not poor (Ref)	—			1.122
	Poor	0.97	0.67–1.41	0.864	
Abuse experience up to age 18	None (Ref)	—			1.141
	Yes	1.09	0.75–1.59	0.653	
Frequency of eating alone	<1 time/week (Ref)	—			1.177
	1–5 times/week	1.41	0.96–2.08	0.084	
	6–7 times/week	0.87	0.56–1.34	0.518	
Habitual use of sleeping pills/anxiolytics	No (Ref)	—			1.030
	Past use	1.97	1.06–3.68	0.033	
	Current use	3.51	2.27–5.44	<0.001	
Family social capital	—	1.01	0.98–1.03	0.715	1.498
UCLA Loneliness Scale (3-item)	—	1.22	1.14–1.30	<0.001	1.342
Risk of social isolation	None (Ref)	—			1.235
	Yes	1.17	0.81–1.68	0.408	
CDC HRQOL-4	Good (Ref)	—			1.048
	Poor	2.92	1.81–4.72	<0.001	
Life satisfaction	—	0.90	0.84–0.97	0.005	1.496

## Data Availability

The data used in the present study are not deposited in a public repository due to containing personally identifiable or potentially sensitive information. In accordance with ethical guidelines in Japan, dissemination of the data is restricted by the Research Ethics Committee of the Osaka International Cancer Institute. Any inquiries regarding data use should be directed to Takahiro Tabuchi (tabuchitak@gmail.com). More details of data availability can be found on the JACSIS website (https://jacsis-study.jp/dug/index.html (accessed on 11 December 2025).
